# Discordant Relationship Between Evaluation of Facial Expression and Subjective Pain Rating Due to the Low Pain Magnitude

**DOI:** 10.29252/NIRP.BCN.9.1.43

**Published:** 2018

**Authors:** Kazuhiro Hayashi, Tatsunori Ikemoto, Takefumi Ueno, Young-Chang Park Arai, Kazuhiro Shimo, Makoto Nishihara, Shigeyuki Suzuki, Takahiro Ushida

**Affiliations:** 1. Multidisciplinary Pain Center, Aichi Medical University, Nagakute, Japan.; 2. Department of Rehabilitation, Nagoya University Hospital, Nagoya, Japan.; 3. Institute of Physical Fitness Sports Medicine and Rehabilitation, Aichi Medical University, Nagakute, Japan.; 4. National Hospital Organization, Hizen Psychiatric Center, Kyushu, Japan.; 5. Program in Physical and Occupational Therapy, Graduate School of Medicine, Nagoya University, Japan.

**Keywords:** Pain assessment, Pain perception, Face

## Abstract

**Introduction:**

Facial expression to pain is an important pain indicator; however, facial movements look unresponsive when perceiving mild pain. The present study investigates whether pain magnitude modulates the relationship between subjective pain rating and an observer’s evaluation of facial expression.

**Methods:**

Twelve healthy volunteers were recruited to obtain 108 samples for pain rating with Visual Analogue Scale (VAS). Subjects underwent three different mechanical painful stimuli (monofilament forces of 100 g, 300 g, and 600 g) over three sessions and their facial expressions were videotaped throughout all sessions. Three observers independently evaluated facial expression of the subjects with a four-point categorical scale (no pain, mild pain, moderate pain, and severe pain). The correlations between subjective pain ratings and the evaluation of facial expression were analyzed in dichotomous group which was low pain ratings (VAS<30), or high pain rating (VAS≥30).

**Results:**

Subjective pain ratings was significantly correlated with the evaluation of facial expression in high pain ratings, however no correlation was found between them in mild pain ratings. In mild pain ratings, most of the subjects (78%) were rated as no pain by observers, despite the fact that subjects reported pain.

**Conclusion:**

The results suggest that the evaluation of facial expression of pain was difficult for the observer to detect pain severity when the subjects feel mild pain.

## 1. Introduction

Subjective pain experience is hard to understand by other people, however, objective pain evaluation is useful to understand pain in someone, especially in patients with communication difficulties (Abbey et al., 2004; Takai et al., 2010; Lord, 2009; Puntillo et al., 1997; Herr et al., 2006). Facial expression responding to pain has emerged as an important objective pain indicator in experimental research as well as in clinical practice (Prkachin, 1992). Moreover, facial expression is considered to be the most prominent way of involuntary communicating affect (Prkachin, 1992; Williams, 2002). It is also a fundamental way of pain communication by displaying and recognizing painful stimuli even in animals (Mogil, 2015). In previous studies, the facial expression is measured using Facial Action Coding System (FACS) (LeResche & Dworkin, 1984), which correlates with subjective pain ratings (Kunz, Mylius, Schepelmann, & Lautenbacher, 2004). However, using FACS requires training and it takes times to become certified as a FACS coder. It includes micro-analytic coding procedures that may be unsuitable or too cumbersome for clinical use. Hence, a simple 4-point categorical scale is commonly used on clinical practice (Abbey et al., 2004; Takai et al., 2010), and such a scale has been believed feasible for evaluating pain in others. Also, a significant correlation is reported between subjective pain, Visual Analogue Scale (VAS), and categorical scale within an individual (Wallenstein, Heidrich, Kaiko, & Houde, 1980; Littman, Walker, & Schneider, 1985).

On the other hand, it has been implied that facial expression was poor in low grade pain (Kunz et al., 2004; Lucey et al., 2012). Kunz et al. have reported that facial expression responding to pain only started when stimulus intensity became strong. Lucey et al. have showed that 62% of subjects were rated as free of pain by observers in spite of feeling pain. These findings have suggested that mild pain is hard to be evaluated by observers. Hence, we speculated that there is a discordant relationship between evaluation of facial expression and subjective pain rating when the subject perceives mild pain. A previous study has reported that when the subjects rated pain score in excess of 30 mm using 100 mm VAS, most of them had recorded at least moderate pain on a 4-point categorical scale (no pain, mild pain, moderate pain, and severe pain) (Collins, Moore, & McQuay, 1997). Therefore, facial expression responding to pain stimulus was expected different after cut-off point of 30 mm on pain-VAS.

The present study investigates the relationships between the evaluation of facial expression responding to pain and self-report ratings in cases where pain rating was categorized as mild pain rating (VAS <30 mm) and moderate to strong pain rating (VAS ≥30 mm). It was hypothesized the relationship between them is weaker in mild pain ratings compared to moderate to strong pain ratings.

## 2. Methods

### 2.1. Subjects

Twelve healthy college student volunteers (7 men and 5 women) participated in this study. Subjects were 21 to 26 years old. All subjects were right-handed, native Japanese speakers, and healthy, without any history of pain or neurological disorders. Ethical approval was obtained from the Research Ethics Committee of the Nagoya University School of Health Sciences. After being informed of the purpose and protocol of the study, all subjects provided written informed consent before undergoing the experiment.

### 2.2. Measurements of pain- Visual Analogue Scale (VAS)

We prepared the self-made Von Frey Monofilament (VFM) for the mechanical stimulating device. The diameter of all VFMs was 1.5 mm and the length of each monofilament (GCK-60® Mitsubishi Reyon Co. Ltd., Japan) was adjusted to produce a different force (100 g, 300 g, and 600 g) because the tissue depth affected by mechanical strain varied depending on the diameter of skin contact (Graven-Nielsen, Mense, & Arendt-Nielsen, 2004; Nasu, Taguchi, & Mizumura, 2010; Takahashi et al., 2005). The examiner practiced many times to ensure that each VFM was successfully applied perpendicular to the target surface until a VFM bending of approximately 3 to 5 mm was produced (Hayashi et al., 2015). All subjects underwent mechanical pain stimuli with von Frey hair filaments (VFHs, diameter: 1.5 mm, strain forces: 100 g, 300 g, and 600 g), on three different sessions once per week. The subjects sat in a fixed chair and placed their right hands open on the desk. Each painful stimulus was given to 3 points of inter-digital sites (second-third, third-fourth, and fourth-fifth finger) of the right hand for 5 s.

Measurements were performed with three different VFMs in 60-s intervals for each stimulus. A curtain in front of the subjects was used to prevent them from viewing the stimulating filaments so as not to predict which kinds of VFM were given during the experiment.

The only information subjects were given was the site of stimulus. The pain-VAS scale consisting of 100 mm lines labeled at the anchor points with “no pain” and “worst possible pain” were measured every time at the end of each stimulus. Measurements were conducted in a quiet room with the temperature kept between 25°C–27°C, and 40%−50% humidity. Videos were taken of subjects facing the front during the experiment. Subjects were only told “videos are taken holistically for our experiment.”

### 2.3. Evaluation of facial expression of pain

We employed 3 evaluators (48-year-old male, 42-year-old-female, and 40-year-old-female) which were healthy and did not have pain. The evaluators had not met subjects before the study and first watched the subjects on video. Facial expression responding to pain was rated by these evaluators using a 4-point categorical scale: 0: No pain; 1: Mild pain; 2: Moderate pain; and 3: Severe pain, referring to the Abbey Scale (Abbey et al., 2004; Takai et al., 2010). The evaluators scored all facial expressions for each subject, and 9 times per subject (100 g: 3 times, 300 g: 3 times, and 600 g: 3 times). Each evaluator independently scored his or her observations. The evaluation was adopted when two of three evaluators gave the same evaluation results. If the evaluations of each evaluator were completely different, the median scale was used.

In addition, weighted kappa statistics were used for the analysis of inter-rater agreement of evaluation for facial expression of pain (Kundel & Polansky, 2003). Weighted kappa statistics were calculated between each pair of evaluators.

### 2.4. Statistical analyses

Normality of the data was assessed by a Shapiro-Wilk test. This test showed that the data of pain-VAS and evaluation of facial expression were not normally disturbed. Therefore, data were expressed as the median and range values, and applied to non-parametric tests. The values of weighted kappa statistics were as follows: virtually no-reliability (0.00–0.10), slight-reliability (0.11–0.40), fair-reliability (0.41–0.60), moderate-reliability (0.61–0.80), and substantial-reliability (0.81–1.00) (Shrout, 1998).

The correlation between the evaluation of facial expression and the self-report pain ratings using pain-VAS were analyzed by using Spearman’s rank correlation coefficient (ρ). Samples with VAS scores less than 30 mm were defined as a low pain group. Those with scores equal to 30 mm or greater were considered to belong to high pain group (Collins et al., 1997; Gerbershagen, Rothaug, Kalkman, & Meissner, 2011; Moore, Straube, & Aldington, 2013).

The correlation between the evaluation of facial expression and VAS was analyzed for each group, respectively. The analyses were performed using SPSS (V. 24.0J; SPSS Inc., Chicago, IL, USA), and the significance was set at P<0.05. Finally, we ran a post hoc power analysis for each analysis using G* Power software (V. 3.0.10; Franz Faul, Kiel University, Kiel, Germany).

## 3. Results

### 3.1. Inter-rater agreement of evaluation of facial expression of pain

One sample (1%) of facial expression were completely different among three evaluators; no pain, mild, and moderate, respectively. It was rated as mild pain. The four level classifications of the evaluation of facial expression of pain showed a high inter-rater agreement both in mild pain ratings (VAS<30) and in strong pain ratings (VAS≥30) (kappa value=0.94–0.95) (Table 1).

**Table 1.** Inter-rater agreement of evaluation for facial expression of pain among 3 evaluators

	**Between 40-Year-Old Female and 42-Year-Old Female**	**Between 40-Year-Old Female and 48-Year-Old Male**	**Between 42-Year-Old Female and 48-Year-Old Male**
Overall (n=108)	0.945	0.949	0.956
VAS<30 (n=37)	0.955	0.958	0.955
VAS≥30 (n=71)	0.941	0.944	0.956

### 3.2. The relationships between self-report pain ratings and the evaluation of facial expression

**Figure 1.**
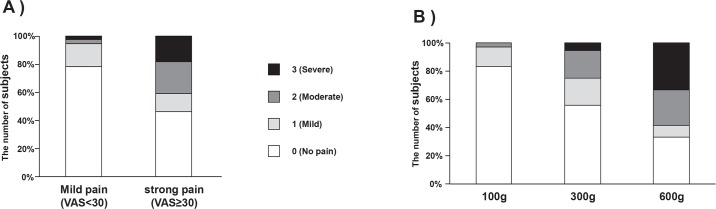
The relationship between the evaluation of facial expression and self-report pain ratings (A) according to self-report pain ratings, and (B) according to pain stimuli intensity

The median (range) values of VAS in overall, in mild pain ratings (VAS<30), and in strong pain ratings (VAS≥30) were 45 (0–84), 12 (0–29), and 59 (30–84), respectively (Table 2). The number of evaluations of facial expression responding to each of pain sensitivities (no pain, mild, moderate, and severe) were 62, 15, 17, 14 as overall; 29, 6, 1, 1 as low pain ratings (VAS<30); and 33, 9, 16, 13 as high pain ratings (VAS≥30); respectively (Figure 1A, Table 2). In low pain ratings (VAS<30), most subjects (78%) were rated no pain by the observers, despite the fact that they reported pain (VAS; 1–29). The number of facial expression rated mild pain or more was 6(16%) in 100 g, 16(44%) in 300 g, and 24(66%) in 600 g (Figure 1B, Table 2).

**Table 2.** The relationship between the evaluation of facial expression and self-report pain ratings

		**Evaluation of Facial Expression**						
		**0 (No pain)**	**1 (Mild)**	**2 (Moderate)**	**3 (Severe)**	**Overall**
Overall (n=108)	No. (%)	62(57)	15(13)	17(15)	14(12)	108(100)
	VAS, median (range)	30(1–69)	42(0–67)	59(26–82)	70(29–84)	45(0–84)
VAS<30 (n=37)	No. (%)	29(78)	6(16)	1(2)	1(2)	37(100)
	VAS, median (range)	12(1–29)	3(0–28)	26(26–26)	29(29–29)	12(0–29)
VAS≥30 (n=71)	No. (%)	33(46)	9(12)	16(22)	13(18)	71(100)
	VAS, median (range)	50(30–69)	56(42–67)	60(38–82)	71(60–84)	59(30–84)
100 g (n=36)	No. (%)	30(83)	5(13)	1(2)	0(0)	36(100)
	VAS, median (range)	13(1–53)	2(0–56)	38(38–38)		12(0–56)
300 g (n=36)	No. (%)	20(55)	7(19)	7(19)	2(5)	36(100)
	VAS, median (range)	44(8–69)	42(27–62)	55(26–63)	44(29–60)	46(8–69)
600 g (n=36)	No. (%)	12(33)	3(8)	9(25)	12(33)	36(100)
	VAS, median (range)	54(18–64)	63(54–67)	63(54–82)	72(60–84)	63(18–84)

VAS: Visual Analogue Scale

Data of VAS are shown as median (range) values. Data of evaluation for facial expression of pain are number of subjects. In mild pain group (VAS<30), most of the subjects (78%) were rated no pain by observers, despite the fact that they felt pain (VAS=1–29).

As shown in Table 3, pain ratings generally showed a moderate correlation (ρ=0.561) with the evaluation of facial expression. Next, further analysis in dichotomous group revealed that while pain ratings significantly correlated (ρ=0.611) with the evaluation of facial expression in high pain ratings; however, there was no significant correlation between them in low pain ratings. Post hoc analysis revealed that they had sufficient statistical power (>80%), respectively (Table 3).

**Table 3.** Correlations between the evaluation of facial expression and self-report pain ratings

	**Correlation Coefficient**	**P**	**Power**
Overall (n=108)	0.561	<0.001	0.999
VAS<30 (n=37)	0.039	0.819	0.823
VAS≥30 (n=71)	0.611	<0.001	0.998

VAS: Visual Analogue Scale

Correlations between the evaluation of facial expression and self-report pain ratings were analyzed by Spearman’s rank correlation coefficient. Self-report pain ratings significantly correlated with the evaluation of facial expression in strong pain ratings (VAS≥30); however, there was no significant correlation between them in mild pain ratings (VAS<30).

## 4. Discussion

Overall, the evaluation of facial expression to pain correlated with self-report pain ratings. However, the correlation was only significant when the subjects were rated as high pain (VAS≥30). In other words, when the subjects were rated as low pain (VAS<30), most of them were rated no pain by observers, despite the fact that they felt pain. The results suggest that it is difficult for observer to find whether the subjects really feel pain or not, when the pain-rating is in low range, and also there is discordant relationship between the evaluation of facial expression responding to pain and self-report pain rating depending on pain magnitude.

It has been reported that self-report pain ratings result in poorer repeatability in mild range compared to high range over different sessions (Hayashi et al., 2015; Quiton & Greenspan, 2008). Kemp et al. has reported that pain-VAS is unreliable in experimental pain when low range or pain threshold (Kemp, Despres, & Dufour, 2012). Regarding the neuronal activities, Hayashi et al. has reported that Blood Oxygenation Level Dependent (BOLD) signal by using functional Magnetic Resonance Imaging (fMRI) was inconsistent in a session with mild pain ratings (Hayashi et al., 2016). The facial expression is related to increase activities in motor-related areas as well as in areas of the thalamocortical pain processing pathways (Kunz, Chen, Lautenbacher, Vachon-Presseau, & Rainville, 2011). These backgrounds might cause discordant relationship between the evaluation of facial expression responding to pain and self-report pain rating in low pain range.

It has been reported that facial expression to painful stimuli is more prominent in strong stimuli compared to weak stimuli (Kunz, Mylius, Schepelmann, & Lautenbacher, 2004; Lucey et al., 2012). Kunz et al. showed only moderate correlation (r=0.4) between self-report pain and facial expression. They have discussed the possibility that stronger stimulus intensity might lead to stronger correlation between them, because low intensities elicited low frequent facial responses (Kunz et al., 2004). They have also reported that facial expression started to appear and increase with stimulus intensities from 4 kg on to the thigh, which corresponded to around 30 mm on VAS (Kunz et al., 2004). While, the present study collected various ratings of pain-VAS from 0 to 84/100 using three different mechanical stimulating forces, the frequency of evaluation as a face of mild pain or more was only 16% in 100 g stimuli, but 44% in 300 g stimuli and 66% in 600 g. It is assumed that these results lead to different correlations between facial expressions and low or high pain ratings, consistent with previous reports (Kunz et al., 2004).

Although the evaluation of facial expression varied slightly among evaluators, there was strong consistency to evaluate categorical scale of pain in the subjects both for low pain ratings and high pain ratings. Someone’s behavioral indicators of pain are usually grimacing, frowning, wincing, vocalization, and restlessness in clinical practice (Puntillo et al., 1997), and each behavior has a potential to be realized by observers as existing pain. However, our experimental results suggest that such behavioral responses may be rarely found when subjects feel mild pain. Hence it is difficult for the observer to find whether the subjects really feel pain or not, when subjective pain-ratings are limited within low range.

Firstly, the facial expression reflects not only pain behavior but also other emotional responses. The facial expression could also be managed, especially by the adult subjects. Although videos were taken of the subjects facing the front with their prior approval during the experiment, subjects were only told that “videos were taken holistically for the purposes of the experiment.” The facial expression responding to pain is influenced by social context (Vlaeyen et al., 2009), sex (Kunz, Gruber, & Lautenbacher, 2006), catastrophizing (Kunz, Chatelle, Lautenbacher, & Rainville, 2008), but not influenced by age (Kunz et al., 2008). Thus, the facial expression responding to pain should be assessed along with such related variables. In addition, the evaluation of facial expression to pain is affected by empathy and sympathy. The evolution of recent cognitive aspects of empathy and sympathy are closely related to processes involved in theory of mind, self-regulation, and language (Decety, 2009).

Secondly, the present study results were not compared with quantitative evaluation of facial expression such as FACS. Whether the findings of the present study are applicable to people of different ethnicities is something which should also be taken into consideration. Finally, we only investigated 12 subjects. A larger study sample might shed more light on these differences.

There is a discordant relationship between the evaluation of facial expression responding to pain and self-report pain rating depending on pain magnitude. The evaluation of facial expression to pain was difficult for the observer when the subjects feel mild pain, even though facial expression is a fundamental way of pain communication. Characteristics of pain evaluation is important for clinical practice.
